# Role of social and other determinants of health in the effect of a multicomponent integrated care strategy on type 2 diabetes mellitus

**DOI:** 10.1186/s12939-020-01188-2

**Published:** 2020-05-24

**Authors:** Rubén Silva-Tinoco, Teresa Cuatecontzi-Xochitiotzi, Viridiana De la Torre-Saldaña, Enrique León-García, Javier Serna-Alvarado, Eileen Guzmán-Olvera, Dolores Cabrera, Juan G. Gay, Diddier Prada

**Affiliations:** 1 Clínica Especializada en el Manejo de la Diabetes de la Ciudad de México-Iztapalapa, Servicios de Salud Pública de la Ciudad de México, Iztapalapa, 09060 Mexico City, Mexico; 2 Servicios de Salud Pública del Gobierno de la Ciudad de México, Mexico City, Mexico; 3 Tecnología e Información para la Salud, TIS, Mexico City, Mexico; 4 Unidad de Investigación Biomédica en Cáncer, Instituto Nacional de Cancerología - Instituto de Investigaciones Biomédicas, Universidad Nacional Autónoma de México, San Fernando 22, Colonia Sección XVI, Tlalpan, 14080 Mexico City, Mexico; 5 Department of Biomedical Informatics, Faculty of Medicine, Universidad Nacional Autónoma de México, Av. Universidad 3000, Circuito Exterior S/N Delegación Coyoacán, 04510 Mexico City, Mexico; 6 Department of Environmental Health Science, Mailman School of Public Health, Columbia University, New York City, 10032 USA

**Keywords:** Effect, Social determinants of health, Multicomponent integrated care strategy, Type 2 diabetes mellitus

## Abstract

**Background:**

Although important advances in treatment strategies have been developed in type 2 diabetes mellitus (T2DM), large gaps exist in achieving glycemic control and preventing complications, particularly in low-and middle-income countries, which suggests a potential effect of social determinants of health (SDH, i.e., education level and socioeconomic status). However, few studies have determined the role of SDH and other determinants of health (ODH, i.e., diabetes knowledge and self-care scores) in achieving T2DM goals during effective multidisciplinary interventions. We aimed to examine a multicomponent integrated care (MIC) program on diabetes care goals and determine the effect of SDH and ODH on T2DM patients.

**Methods:**

A before-and-after design (a pretest, a 5-month intervention, and a follow-up) was used in a T2DM population from Mexico City. The SDH included education level and socioeconomic status; the ODH included diabetes knowledge, self-care scores, and deltas (i.e., differences between baseline and follow-up scores). The triple-target goal (glycated hemoglobin, blood pressure, and LDL-cholesterol) was established as a measurement of T2DM goals.

**Results:**

The DIABEMPIC (DIABetes EMPowerment and Improvement of Care) intervention (*n* = 498) reduced the glycated hemoglobin levels (mean reduction 2.65%, standard deviation [SD]: 2.02%) and cardiometabolic parameters; it also improved health-related quality of life. From 1.81% at baseline, 25.9% of participants (*p-*value< 0.001) achieved the triple-target goal. We found a significant association between education level (*p*-value = 0.010), diabetes knowledge at baseline (*p*-value = 0.004), and self-care scores at baseline (*p*-value = 0.033) in the delta (change between baseline and follow-up assessments) of HbA1c levels. Improvements (increase) in diabetes knowledge (*p*-value = 0.006) and self-care scores (*p*-value = 0.002) were also associated with greater reductions in HbA1c.

**Conclusions:**

MIC strategies in urban primary care settings contribute to control of T2DM. SDH, such as education level, and ODH (diabetes knowledge and self-care scores at baseline) play a key role in improving glycemic control in these settings.

## Introduction

Diabetes mellitus is a chronic metabolic disease with disabling, deadly, and costly consequences for individuals, families, and national health care systems. Proper diabetes management in people living with type 2 diabetes mellitus (T2DM) focuses on reducing the risks for macrovascular and microvascular complications by controlling blood pressure, lipid levels, and blood glucose levels, and by avoiding tobacco [1]. Although important advances in pharmacological and non-pharmacological strategies have been developed, large gaps exist in achieving care goals, particularly in real-world practice and low- and middle- income countries (LMICs) [2–4]. Barriers to achieving care goals in T2DM patients may include lack of medical care, poverty, long commutes or lack of time to seek medical attention, lack of confidence, and inadequate social support, among others [5]. Achieving diabetes care goals (e.g., the triple-target goal [6]: normal glycated hemoglobin, blood pressure, and LDL cholesterol) is associated with better health outcomes, including lower risks of complicating events and death [6, 7].

Social determinants of health (SDH), including education level and socioeconomic status (SES), as well as other key factors (diabetes knowledge and self-care scores), named here as other determinants of health (ODH), have demonstrated to modify the response to therapy in T2DM patients [8–10]. In a previous study, we reported the baseline determinants of glycemic control in T2DM patients and public primary care in Mexico City [11]. It has been suggested that in LMICs urban settings, where the burden of the disease is particularly high, urgent improvements in health care systems are needed to reduce complications and mortality [12, 13]. Diabetes multicomponent programs include strategies for quality on diabetes care improvement directed to patients, health care professionals or systems, particularly focused on enabling periodic evaluations of quality-of-care indicators to identify treatment gaps and disseminate information to promote better decision-making processes [13]. Previous research has shown important improvement in achieving diabetes care goals through multicomponent quality improvement strategies [13, 14]. However, real-world evidence of primary care experiences in public health systems in LMICs is scant for research purposes [13, 15]. The evidence is even scanter for the study of the effect of SDH and ODH on achieving diabetes care goals [15].

Considering these gaps, the purposes of this study were (1) to describe the effect of a diabetes multicomponent integrated care (MIC) program on diabetes care goals (glycated hemoglobin, blood pressure, and LDL cholesterol) and (2) to examine the effect of SDH (education level and SES) and ODH (diabetes knowledge, self-care scores) on T2DM. We aimed to determine the role of SDH and ODH in the context of a multi-intervention program (DIABEMPIC, DIABetes EMPowerment and Improvement of Care), using a before-and-after design and testing the effect of baseline SDH and ODH in the delta (Δ) of change in HbA1c levels.

Mexico City (MC) is one of the largest cities in the world; it is highly polluted and has a high prevalence of obesity, T2DM, and metabolic syndrome. Although ethnically homogeneous, MC also shows wide socioeconomic disparities, with more than 680,000 people living under extreme poverty [16], having low levels of education, lacking health insurance, and constantly exposed to social and domestic violence. Therefore, MC is a strategic place to determine the effects of SDH and ODH on T2DM by using pharmacologic and non-pharmacologic interventions such as DIABEMPIC.

## Subjects

All T2DM patients came from 32 primary outpatient health care centers located in urban areas of Mexico City and were referred to the *Clínica Especializada en Diabetes CDMX/Iztapalapa* (Specialist Diabetes Clinic) between January 2017 and July 2018. They were invited to participate in the 5-month *DIABEMPIC (DIABetes EMPowerment and Improvement of Care)* program, which is a primary care strategy of the public primary health care system, designed to improve clinical outcomes in T2DM patients through interdisciplinary care and self-management education schemes. The participation criteria were as follows: (a) T2DM patients older than 18 years, (b) without any acute or chronic complication that required short-term hospital care, and (c) acceptance to participate after understanding the program.

## Materials and methods

### Design and population

We made a before-and-after design to determine the effect of the DIABEMPIC intervention on diabetes care goals. We recorded SDH and ODH at baseline and after the 5-month intervention. In strict adherence to the Declaration of Helsinki and the Good Clinical Practice, we obtained the approval from the Institutional Review Board (609–010–01-18), and all participants provided verbal and written informed consent. This study was registered in ClinicalTrials.gov (Identifier: NCT04245267).

### Assessment of clinical and socioeconomic factors, diabetes knowledge, self-care activities, and health-related quality of life

Data were collected during medical interviews and included demographic characteristics, current treatment, time since diagnosis, and comorbidities. The staff that performed the initial assessment of the patients (endocrinologists) was different from the one who provided the intervention (interdisciplinary care team). We also collected information about physical examination, weight, blood pressure, and biochemical data, including glycated hemoglobin (HbA1c) and LDL cholesterol (LDL-C) as a measure of glycemic control and lipid control, respectively. The socioeconomic status was determined using the AMAI index (Spanish for Mexican Association of Marketing Research and Public Opinion Agencies*)* [17], which integrates updated information on income and expenses of Mexican households from official government databases. The index generates a numeric value (0 to > 193) and five categories ranging from “A/B” category, the highest socioeconomic level, to “E” category, the lowest one [15]. Diabetes knowledge was assessed using the Spoken Knowledge in Low Literacy Patients with Diabetes (SKILLD) scale [18]. The 10-item SKILLD assesses the knowledge of lifestyle interventions, glucose management, recognition and treatment of hypo- and hyperglycemia, and activities to prevent long-term diabetes-related complications (the sum of the score ranged from 0 to 10). The SKILLD was initially designed and validated for vulnerable T2DM patients with low literacy, and it has been previously used in Mexican-origin populations [19–21]. The 11-item version of the Summary of Diabetes Self-Care Activities (SDSCA) [22] was used to measure the frequency of self-care behavior in the last 7 days. In the analyses, we included general diet, specific diet (fruits/low-fat diet), exercising, glucose testing, and foot care. Participants were asked to rate their health-related quality of life (using the EuroQol-5D-5 L visual analog scale, with scores ranging from 0 to 100) [23].

### Intervention: the DIABEMPIC program

DIABEMPIC is a 5-month interdisciplinary and empowerment-based program that includes individual and group sessions in a scheme of ambulatory, scheduled, and shared medical appointments. The multidisciplinary and case management team involves an endocrinologist or diabetologist, a nutritionist, a diabetes-trained nurse, a psychologist, a social worker, a podiatrist, and an ophthalmologist. The components of the DIABEMPIC program are diverse and include a case management team, a diabetes self-management education program, adequate consultation time (30–45 min), audit and feedback, guaranteed medication supply, high-quality electronic records, attention to different components on the same day, visit planning, and short-term follow-up. The DIABEMPIC intervention consists of 26 h, distributed through a 5-month program. The topics of the educational sessions include general knowledge about diabetes, self-identification of diabetes care goals, reduction of risks and diabetes-related complications, healthy food preparation and combinations, physical activity and exercise, and myths and realities about diabetes. Our team implemented quality control (QC) and quality assurance (QA) for all the components, with continuous monitoring of activities, and specialized patient handling in each step of the program. QC and QA included daily reviews of the patients coming to the clinic, verification of compliance to indications, attendance to individual and group sessions, among others. In case of deviations, appropriate measures were taken. To effectively reach a low-literate audience, we used simple language and pictorial aids. Using quality-of-care indicators for health-care professionals, a medical coordinator conducted an audit and gave feedback regarding compliance and effectiveness. The interventions, including laboratory tests, were free to the patients. Medication supply for glycemic control (metformin, dipeptidyl peptidase-4 inhibitors, sulfonylureas, human insulin, and insulin analogs) was guaranteed and covered by the health care system.

### Statistical analysis

We determined the effect of the intervention on the improvement in metabolic parameters (glycemic control, blood pressure, LDL-C, weight, and body mass index) as well as in diabetes knowledge, self-care activities (diet, physical activity, glucometer readings, foot care, and global self-care), and quality of life using a before-and-after design [24], including mean and 95% confidence intervals for the baseline and 5 months after recruitment. The differences between baseline and after the intervention were determined using non-parametric tests (Wilcoxon test). We also compared the proportion of patients reaching the triple target (HbA1c < 7%, blood pressure < 130/80 mmHg, and LDL-C < 100 mg/dL) at baseline, as well as after the intervention. The differences were established using a chi-squared test. To determine the effect of SDH and ODH on the intervention, we first obtained delta (Δ) of change for the most relevant outcomes (glycemic control, blood pressure, LDL-C, weight, and body mass index) as well as for the intervention (diabetes knowledge and self-care). We explored the role of socioeconomic determinants (education and socioeconomic status) on the Δ of change, using unadjusted and multivariable linear logistic regression models. Statistical significance was defined as a value of *p* <  0.05. The analyses were done using the R software (R Project for Statistical Computing, CRAN, The Comprehensive R Archive Network, Vienna).

## Results

### Characteristics of the study participants

We included 498 patients with type 2 diabetes. All participants concluded the 14 h of the multidisciplinary team intervention and the 12 h of the diabetes self-management education program, for a total of 26 h. Most of them were women (65.66%). The mean age was 54.88 years (standard deviation [SD]: 11.01 years). More than half (53.01%) had completed primary school or less, and 56.83% had low or very low socioeconomic status (D+, D, or E, AMAI score categories). T2DM patients showed a mean of 12.05 years since diagnosis (SD: 8.23 years) and a high frequency of microvascular complications, especially distal neuropathy (49.40%) and kidney disease (48.19%). They also showed a mean HbA1c baseline value of 9.5% (80 mmol/mol). The full description of the population included is shown in Table 1.

**Table 1:** Sociodemographic and clinical characteristics at baseline of patients with type 2 diabetes mellitus (*n* = 498)

Continuous variables	Mean	SD
Age (years)	54.88	11.01
Years since diagnosis	12.05	8.23
Socioeconomic status (score)^a^	85.83	51.61
HbA1c (%)	9.48	2.16
**Categorical variables**	**n**	**%**
**Sex**		
Female	327	65.66%
Male	171	34.34%
**Education**		
Null	25	5.02%
Cannot read nor write	52	10.44%
Primary school	187	37.55%
Junior high	117	23.49%
High school	72	14.46%
University	37	7.43%
No information	8	1.61%
**Socioeconomic status**		
A, B (> 193)	8	1.61%
C+ (155 to 192)	23	4.62%
C (128 to 154)	42	8.43%
C- (105 to 127)	53	10.64%
D+ (80 to 104)	105	21.08%
D (33 to 79)	163	32.73%
E (0 to 32)	15	3.01%
No information	89	17.87%
**Comorbidities**		
Hypertension	269	54.02%
Hypertriglyceridemia	291	58.43%
Hypercholesterolemia	253	50.80%
**Microvascular complications**		
Diabetic retinopathy	135	27.11%
Diabetic kidney disease	240	48.19%
Distal diabetic neuropathy	246	49.40%

^a^Score for socioeconomic status. *SD* Standard deviation

### DIABEMPIC intervention was associated with improvement in diabetes outcomes

**Fig. 1:**
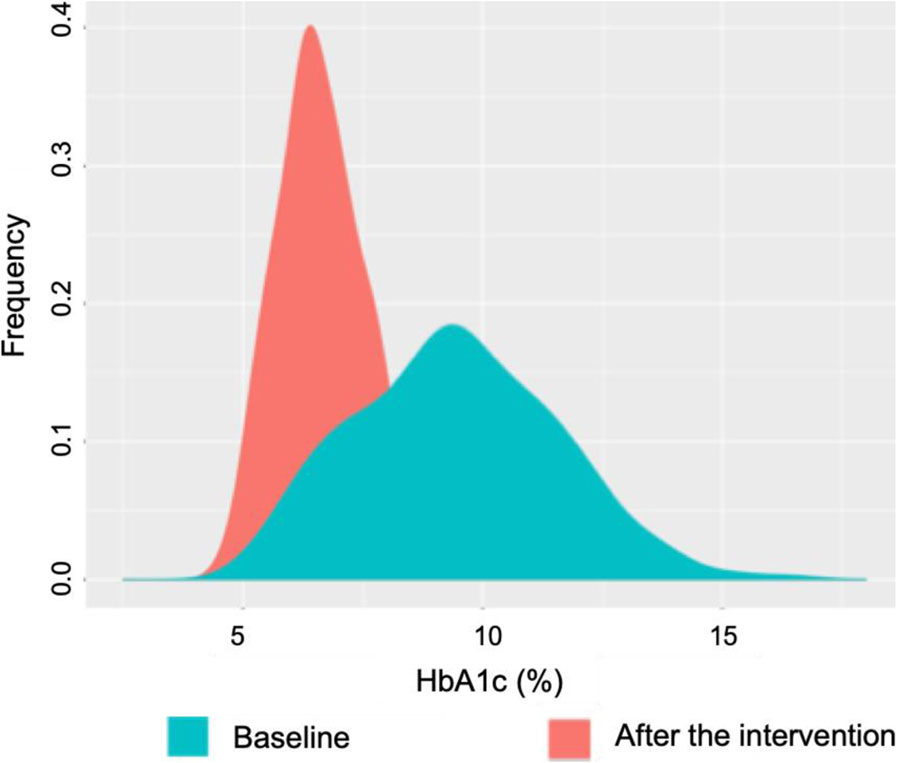
Kernel representation of HbA1c levels (%) at baseline and after the DIABEMPIC intervention in type 2 diabetes mellitus patients (*n* = 498)

We observed a statistical significance between baseline and after the intervention for all the evaluated diabetes care goals (Table 2). We observed a mean reduction in HbA1c (2.65%), systolic (9.7 mmHg) and diastolic (3.23 mmHg) blood pressure, and LDL-C (18.2 mg/dL). Weight and body mass index (BMI) also showed a moderate but significant reduction (1.6 kg and 0.6 kg/m^2^, respectively) after the intervention. The mean and 95% confidence intervals for metabolic parameters evaluated in the study are shown in Table 2. The Kernel diagram of HbA1c levels allowed us to detect the tendency of returning to the mean after the intervention, in comparison with the baseline values (Fig. 1).

**Table 2:** Mean and 95% confidence interval for clinically relevant variables at baseline and after intervention in patients with type 2 diabetes mellitus (*n* = 498)

Variable	Baseline		After intervention		*p*-value
	Mean	95% CI	Mean	95% CI	
**Triple target**					
Glycemic control (HbA1c1, %)	9.48	(9.285, 9.666)	6.83	(6.720, 6.935)	**< 0.001**
Blood pressure (mm Hg)					
Systolic	128.79	(127.034–130.552)	119.11	(117.735, 120.488)	**< 0.001**
Diastolic	74.21	(73.355, 75.070)	70.99	(70.196, 71.780)	**0.003**
LDL-Cholesterol (mg/dL)	111.10	(107.667, 114.528)	92.89	(89.976, 95.803)	**< 0.001**
**Diabetes knowledge**	3.06	(2.848, 3.265)	8.06	(7.901, 8.223)	**< 0.001**
**Self-care activities**					
Diet					
Specific	2.92	(2.764, 3.067)	4.39	(4.236, 4.539)	**< 0.001**
Global	2.46	(2.278, 2.648)	5.71	(5.575, 5.838)	**< 0.001**
Total	2.69	(2.549, 2.829)	5.05	(4.933, 5.162)	**< 0.001**
Physical activity	1.85	(1.650, 2.051)	4.09	(3.897, 4.290)	**< 0.001**
Glucometer	1.66	(1.456, 1.854)	4.01	(3.840, 4.176)	**< 0.001**
Foot care	3.52	(3.245, 3.791)	6.63	(6.527, 6.728)	**< 0.001**
Self-care global score^a^	2.42	(2.287, 2.559)	4.92	(4.828, 5.017)	**< 0.001**
**Other variables**					
Weight (kg)	73.19	(71.783, 74.590)	71.56	(70.215, 72.905)	**< 0.001**
BMI (kg/m^2^)	30.24	(29.693, 30.797)	29.57	(29.048, 30.092)	**< 0.001**
Quality of life by VAS	60.61	(58.583, 62.633)	86.97	(85.935, 88.008)	**< 0.001**
	n	%	n	%	
**Proportion of patients per achieved goal**					
Glycemic control (HbA1c1, %)	64	12.85%	313	62.85%	**< 0.001**
Blood pressure (mm Hg)					
Systolic	287	57.63%	362	73.43%	**< 0.001**
Diastolic	363	72.89%	394	80.08%	**0.009**
LDL-Cholesterol (mg/dL)	160	39.41%	281	65.50%	**< 0.001**
Triple target	9	1.81%	129	25.90%	**< 0.001**

95% *CI* 95% Confidence interval. ^a^Diet, physical activity, glucose, and foot care. *VAS* Visual analogue scale. *BM*I Body mass index. *LDL* Low density lipoproteins

### DIABEMPIC intervention positively modified diabetes knowledge, self-care activities, and health-related quality of life

To determine the intensity of changes in diabetes knowledge, self-care, and health-related quality of life (HRQOL) after the intervention, we compared these factors at baseline and after the intervention. We found statistically significant changes for all the evaluated variables. We observed an increase of 5.01 in the score of diabetes knowledge, which represented an increase of 163.79% from baseline. The frequency (days a week [d/w]) of performance of self-care activities improved in all the evaluated parameters, including specific diet (an increase of 1.47 d/w, improvement of 50.48% from baseline), global diet (an increase of 3.24 d/w, improvement of 131.72% from baseline), physical activity (an increase of 2.24 d/w, improvement of 121.22% from baseline), foot care (an increase of 3.11 d/w, improvement of 88.38% from baseline), among others. We also observed an improvement in the health-related quality of life rate (an increase of 26.36 points, improvement of 43.50% from baseline), determined by the EuroQol-5D-5 L visual analog scale. Full description for the mean values and 95% confidence intervals for knowledge, self-care in DM, and quality of life before and after the intervention is shown in Table 2.

### The DIABEMPIC intervention contributed to reaching the triple target

We also obtained statistically significant differences between baseline and after the intervention in relation to cardiometabolic targets (HbA1c < 7%, blood pressure < 130/80 mmHg, and LDL-C < 100 mg/dL), both individually and as an integrated outcome (the three outcomes reached). The intervention had the strongest impact on the integrated target: more people reached the three outcomes, from 1.81% at baseline to 25.90% after the intervention. Individually, glycemic control (HbA1c < 7%) increased from 12.85 to 62.85%, that is, 4.9 times higher in comparison with the baseline values. Although modest, the differences observed in blood pressure and LDL-C were statistically significant. The full description of the variables studied and the triple-target frequency, individually and integrated, before and after the intervention, is shown in Table 2.

### Role of sociodemographic factors, diabetes knowledge, and self-care at baseline on diabetes improvements after DIABEMPIC

Finally, we explored the role of SDH and ODH in the delta of change in the markers of cardiometabolic improvement (delta-HbA1c, delta-blood pressure, delta-cholesterol, and integrated triple target) using unadjusted and multivariable-adjusted models. We found a significant association between the education level in the delta of HbA1c levels in unadjusted (*p*-value = 0.014) and in multivariable-adjusted models (*p*-value = 0.010, Table 3). We also found a positive correlation between diabetes knowledge at baseline and the delta of HbA1c (*r* = 0.13, *p*-value = 0.004), which suggests that lower baseline diabetes knowledge may predict greater improvements in HbA1c. This finding was also confirmed in unadjusted models (*p*-value = 0.004) and in multivariable-adjusted models (*p*-value = 0.004, Table 3). A weak correlation, but still statistically significant, was observed for self-care scores at baseline in HbA1c (*r* = 0.09, *p*-value = 0.032) and confirmed in unadjusted models (*p*-value = 0.033) and in multivariable-adjusted models (*p*-value = 0.033, Table 3), which indicates greater benefits in HbA1c levels in those patients with lower baseline self-care scores. We also observed an association between education level at baseline and the delta of systolic blood pressure (*p*-value =0.027), but this association was lost in multivariable-adjusted models (*p*-value = 0.093, Supplementary Table 1). We also checked for a potential interaction of SES and education in the association between the intervention and outcome, but our data did not meet the assumptions for an ANCOVA test (no linear relationships between pre and post-HbA1c, no normality of residuals, and presence of relevant outliers), suggesting a lack of interaction.

**Table 3:** Association between social and other determinants of health (SDH and ODH) in the delta of change in HbA1c1 in DIABEMPIC participants (*n* = 498)

	Unadjusted			Multivariable^b^		
	β	95% CI	*p*-value	β	95% CI	*p*-value
**Δ-HbA1c**						
Education^a^	1.025	(0.213, 1.838)	**0.014**	1.092	(0.267, 1.916)	**0.010**
SES	0.001	(−0.004, 0.005)	0.736	0.001	(−0.004, 0.005)	0.807
Diabetes Knowledge (baseline)	0.111	(0.037, 0.186)	**0.004**	0.113	(0.036, 0.190)	**0.004**
Diabetes Knowledge (final)	−0.004	(− 0.101, 0.094)	0.937	0.001	(−0.100, 0.101)	0.992
Self-care (baseline)	0.126	(0.011, 0.240)	**0.033**	0.126	(0.011, 0.242)	**0.033**
Self-care (final)	−0.156	(−0.322 0.009)	0.064	−0.154	(−0.320, 0.012)	0.069
Δ-knowledge	−0.107	(−0.180, − 0.035)	**0.004**	−0.102	(− 0.176, − 0.029)	**0.006**
Δ-self-care	− 0.166	(− 0.270, − 0.063)	**0.002**	−0.165	(− 0.269, − 0.061)	**0.002**

^a^Dichotomized (null vs the rest of categories). *SES* Socioeconomic status. ^b^Models adjusted by age (continuous), sex (categorical) and years of disease (continuous). 95% *CI* 95% Confidence interval. *DM* Diabetes mellitus. Δ-HbA1c: Difference between the first and the last HbA1c

### Role of diabetes knowledge and self-care improvements in diabetes improvements after DIABEMPIC

**Fig. 2:**
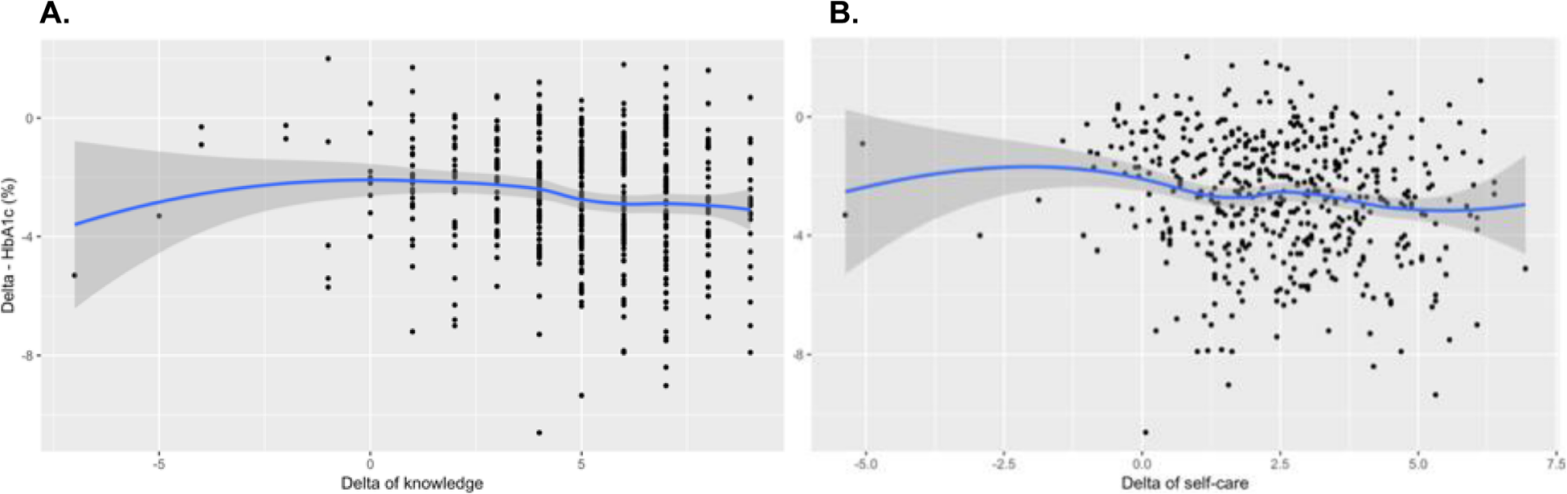
Smoothed conditional means for the linear association between: **a**. The Δ of diabetes knowledge (Δ-DK, difference between DK after the intervention minus the baseline score for DK), and **b**. The Δ of diabetes self-care (Δ-DSC, difference between DSC after the intervention minus the baseline score for DSC) in HbA1c in type 2 diabetes mellitus patients (*n* = 498)

We found a negative correlation between the delta of change for diabetes knowledge in the delta of HbA1c (*r* = − 0.13, *p*-value = 0.004), which suggests that greater improvements in diabetes knowledge would contribute to a stronger impact on the reduction of HbA1c. This finding was confirmed in multivariable-adjusted models (*p*-value = 0.006, Fig. 2, Panel a). A similar trend was observed in the impact of the delta of changes in self-care scores on the delta of HbA1c (*r* = − 0.14, *p*-value = 0.002), which suggests that a greater improvement in diabetes self-care contributes to a stronger impact on the reduction of HbA1c. This finding was also confirmed in multivariable-adjusted models (*p*-value = 0.002, Fig. 2, Panel b). We did not find any effect of socioeconomic status, diabetes knowledge final score, or self-care final score on the delta of HbA1c. A full description of the interactive factors in the delta for HbA1c levels is shown in Table 3. A similar approach was used to determine the effect of sociodemographic factors, diabetes knowledge, and self-care in diabetes on the delta of blood pressure and LDL cholesterol; however, few associations were observed (Supplementary Table 1). We also explored the potential effects of these factors in the triple target, both at the baseline and at the end of the intervention, but no statistical significance was observed (Supplementary Table 2). We did not observe associations in the triple target in unadjusted models that were lost after multivariable adjustment (Supplementary Table 2).

## Discussion

This study aimed to analyze the effect of a MIC intervention on diabetes care goals and to explore potential effects of SDH and ODH on T2DM patients with adverse social characteristics in an urban primary care setting. The DIABEMPIC program significantly improved glycemic control, blood pressure, LDL-C, weight, and HRQOL; it also helped to achieve the diabetes care targets, both separately and combined. We also found that the positive impact for glycemic control was greater among T2DM patients with the lowest education levels and those with the lowest baseline diabetes knowledge and self-care scores, but also among those with greater improvements of diabetes knowledge and self-care activities scores after the intervention. To the best of our knowledge, this is the first study that explores the effect of a MIC intervention and addresses multiple barriers to care in T2DM patients with a predominance of low schooling and low socioeconomic status in a public primary health care system in Mexico. Additionally, to our knowledge, this is one of the largest studies that tries to elucidate the role and contribution of SDH and ODH and clinical determinants of health in the context of a quality improvement intervention in diabetes care goals in an LMIC. A relevant fact is that all the included patients in this study were beneficiaries of *Seguro Popular (now INSABI),* the national health care system attending the largest proportion of primary care outpatients in Mexico [25]. These patients belong to a population that traditionally lacks formal social and health insurance.

The results of glycemic control shown in this study are more substantial than previously reported [13–15]. This size effect could be explained by some potential factors: it was previously reported that MIC programs improved clinical outcomes, particularly in young T2DM patients, with suboptimal control and in low-resource settings [13]. The DIABEMPIC program included multiple and different strategies that have demonstrated positive results in diabetes outcomes and surrogate endpoints, all of them integrated into one place. The integration of multiple strategies into an organized way is difficult to occur in real-world practice because it involves health care systems, health care professionals, and T2DM patients. We consider that we have explored the effect of a quality improvement strategy in T2DM patients who had participated in few- or none- of the strategies included in the program, partly due to social backwardness. Thus, we are observing a multiplier effect in a population that is almost unaware of such strategies. To support this factor, we previously reported that poor education levels were linked to poor diabetes knowledge and poor glycemic control [11].

In this study, we observed that the greatest improvements in glycemic control occurred in T2DM patients with the greatest improvements in self-care activities performance and diabetes knowledge. Our results strengthen the recommendation to integrate structured therapeutic education programs linked to improving quality strategies because their impact could be even greater on socially disadvantaged populations. Poverty influences the development of type 2 diabetes and its complications [26]; education and socioeconomic levels are associated with activating self-care management in chronic diseases [27]. Thus, multicomponent quality improvements of care favor narrowing health and social gaps in T2DM patients, as well as some lagging indicators such as poor education level and health education. An even more important fact is the possibility to improve long-term outcomes, where structured therapeutic patient education strategies integrated into MIC could play a decisive role [28].

Despite the important improvements in diabetes care goals, a proportion of T2DM patients did not reach the 3 goals targets set in this study. However, the study population had certain characteristics that prevented some participants from reaching the targets; for instance, the long-term diabetes diagnosis and potential risk for hypoglycemia/hypotension made them unsuitable for achieving ambitious care goals [29].. Compared with 1% of Mexican male patients and 12% of Americans [30], 25.9% of our study participants reached the three control goals.

In T2DM patients with poor self-care, perhaps conditioned to some degree by lack of knowledge, implementing diabetes self-management education (DSME) programs favors improving different interrelated activities performed by patients, including adhering to pharmacological and non-pharmacological recommendations [28]. The latter implies that multiple diabetes care goals beyond glycemic control, such as lipid and blood pressure control and weight reduction, are more likely to occur as a product of a stricter adherence and lifestyle modifications. An integrated team of health care professionals (HCPs) in a shared medical appointment model not only allows HCPs to personalize recommendations but also to strengthen health literacy and skills through a strategy of multiple same-day messages delivered by HCPs. This strategy facilitates creating shared decision plans and allows for follow-ups and feedback among T2DM patients and the team of HCPs. These care schemes have previously demonstrated to decrease HbA1c [31], LDL-C, and systolic blood pressure [32].

In terms of generalizability, our patients had characteristics particular to Latin-Americans with T2DM, including low-income and mixed genetic background. In terms of sex distribution, our study showed a higher number of women than men. This finding does not agree with the most recent National Health and Nutrition Survey – Mexico, which showed an equal sex distribution among T2DM in this age group [25]. This finding may suggest a potential population bias indicating that women tend to seek medical attention more often. Further research on how sex influences seeking medical attention in T2DM patients in Mexico City is warranted.

This study has several limitations. This is a before-and-after study, and individuals may not be comparable in their demographics and characteristics after 5 months. However, the strategies used in this study have been widely proven to be effective and are part of the current standards of care [33–35]. Thus we reported real-world experiences in a public health care system providing services in a middle-income country. Also, the program results may not translate into long-term sustainability. Nevertheless, some studies have demonstrated that when implementing strategies similar to ours, long-term benefits are obtainable, including decreasing the risks of microvascular and macrovascular complications [36, 37] and all-cause mortality [38], independently of maintaining diabetes care goals, even if the immediate effect on diabetes care goals is minimal [39]. Moreover, patient empowerment and quality improvement interventions have demonstrated to be cost-effective in long-term empirical estimates [40, 41]. Additionally, despite the important improvements in diabetes care goals, a proportion of T2DM patients did not reach the target goals set in this study. However, the study population had certain characteristics that made these targets unreachable for some patients; for instance, the long-term diabetes diagnosis and the potential risk for hypoglycemia/hypotension made participants unsuitable for achieving ambitious target goals [29]. On the other hand, this study was conducted in Mexico City, a megapolis, and it is possible that our findings are not representative of other urban settings across the world. Nevertheless, the study highlights the need of considering SDH and ODH across populations that surely share similarities with our sample. Also, different factors regarding diabetes care goals achievement were not analyzed. However, diabetes care goals and diabetes-related outcomes have complex and multicausal origins, including biological, individual, and social factors. Our results are potentially affected by self-selection bias, particularly due to patients that did not agree to participate during the initial recruitment process or who were not interested in improving their diabetes management or outcomes. However, patients referred to the DIABEMPIC program came from primary level hospitals, and most of them agreed to participate and follow this program because of its free access and long-term health care benefits. 88% of the requested to participate accepted to follow the program and the rates of long-term participation in the program were 88%. Although we did not quantify the number of individuals included in this potential self-selection bias, our team reduced the possibilities of such bias: they made a remarkable effort to motivate and follow up all potential participants by making them and their family aware of the medical, economic, and social consequences of improper diabetes management. We also recognize that our study is limited by its design (uncontrolled before-and-after design). The limitations of our design include a lack of a control group and, therefore, a lack of randomization. However, we could not include a control group for ethical reasons (i.e., all the DIABEMPIC interventions have previously demonstrated a benefit in T2DM), and as the same individuals are followed after the intervention, this design is considered experimental [24]. Also, significant differences observed between time points in the outcomes may not have resulted from the intervention but might have been due to confounding. Even though the statistical analyses were adjusted for confounding by known variables, we recognize that it was not possible to record all characteristics that may influence the association between the intervention and outcome measures. Additionally, our study lacks a control group to determine the effect of the intervention, as well as the effect of SDH and ODH in this context. However, the components of the intervention program had an independent positive effect on diabetes clinical goals, including HbA1c. Therefore, to compare the intervention in a randomized study would fall within the ethical sphere of T2DM therapy.

## Conclusions

Our findings support an encouraging proposal to address the overwhelming disease burden related to diabetes care, especially in populations with adverse SDH and ODH, where the implementation of quality improvement strategies probably lacks, but also where health equity in diabetes care can be achieved through such strategies. Long-term sustainability and cost-effective analysis are necessary, as well as quality of life and satisfaction of T2DM patients participating in these strategies. Diabetes care demands changing the role perspective of health care systems, HCPs, and T2DM patients to improve diabetes-related outcomes. MIC strategies could help to balance the co-responsibility, but the quality of care must be first assured in a favorable medical environment. Multicomponent initiatives with potential effectiveness in LMICs in T2DM must be replicated because they may have positive implications for population health and health care costs.

## Supplementary information

**Additional file 1: Table S1.** Association between social determinants of health, diabetes knowledge, and self-care in the delta of change in blood pressure and LDL-Cholesterol levels in DIABEMPIC participants (*n* = 498).

**Additional file 2: Table S2.** Association between social determinants of health, diabetes knowledge, and self-care in successfully reaching the triple target in DIABEMPIC participants (*n* = 498).

## Data Availability

The datasets generated and/or analyzed during the current study are not publicly available because the data belong to patients from the Clinic and are under confidentiality policies, but are available from the corresponding author on reasonable request and by using unidentifiable IDs by a third party.
