# Supplementary material for: Role of social and other determinants of health in the effect of a multicomponent integrated care strategy on type 2 diabetes mellitus
Source: Int J Equity Health. 2020 May 24;19:75. doi: 10.1186/s12939-020-01188-2 (PMC7245830; doi:10.1186/s12939-020-01188-2)
Supplement: Supplementary file 1 — Additional file 1: Table S1. Association between social determinants of health, diabetes knowledge, and self-care in the delta of change in blood pressure and LDL-Cholesterol levels in DIABEMPIC participants (n = 498). [file 12939_2020_1188_MOESM1_ESM.docx]

| **Supplementary Table 1.** Association between social determinants of health, diabetes knowledge, and self-care in the delta of change in blood pressure and LDL-Cholesterol levels in DIABEMPIC participants (n=498). | | | | | | | | | |
| --- | --- | --- | --- | --- | --- | --- | --- | --- | --- |
|  |  | Univariable | | |  | Multivariable | | | |
|  |  | β | 95% CI | *p*-value |  | β | 95% CI | *p*-value |  |
| **Blood pressure** | |  |  |  |  |  |  |  |  |
| Δ-Systolic | |  |  |  |  |  |  |  |  |
|  | Education level* | 4.944 | (-2.461, 12.349) | 0.191 |  | 2.597 | (-4.804, 9.998) | 0.492 |  |
|  | SES | 0.040 | (-0.002, 0.081) | 0.063 |  | 0.042 | (0.001, 0.083) | **0.048** |  |
|  | Diabetes knowledge (baseline) | -0.028 | (-0.705, 0.648) | 0.935 |  | -0.288 | (-0.975, 0.399) | 0.412 |  |
|  | Diabetes knowledge (final) | 0.389 | (-0.484, 1.262) | 0.383 |  | -0.045 | (-0.932, 0.841) | 0.920 |  |
|  | Self-care (baseline) | 0.856 | (-0.173, 1.885) | 0.104 |  | 0.707 | (-0.315, 1.729) | 0.176 |  |
|  | Self-care (final) | 0.025 | (-1.468, 1.518) | 0.974 |  | -0.168 | (-1.643, 1.307) | 0.824 |  |
|  | Δ-knowledge | 0.246 | (-0.410, 0.901) | 0.463 |  | 0.234 | (-0.417, 0.885) | 0.482 |  |
|  | Δ-self-care | -0.706 | (-1.647, 0.236) | 0.142 |  | -0.653 | (-1.583, 0.277) | 0.170 |  |
| Δ-Diastolic | |  |  |  |  |  |  |  |  |
|  | Education level* | 4.710 | (0.559, 8.861) | **0.027** |  | 3.571 | (-0.589, 7.732) | 0.093 |  |
|  | SES | 0.022 | (-0.001, 0.046) | 0.066 |  | 0.022 | (-0.001, 0.046) | 0.063 |  |
|  | Diabetes knowledge (baseline) | 0.059 | (-0.322, 0.440) | 0.761 |  | -0.086 | (-0.475, 0.302) | 0.663 |  |
|  | Diabetes knowledge (final) | 0.211 | (-0.281, 0.702) | 0.401 |  | 0.010 | (-0.491, 0.512) | 0.967 |  |
|  | Self-care (baseline) | -0.285 | (-0.866, 0.296) | 0.337 |  | -0.370 | (-0.948, 0.209) | 0.211 |  |
|  | Self-care (final) | -0.044 | (-0.886, 0.797) | 0.918 |  | -0.110 | (-0.944, 0.724) | 0.797 |  |
|  | Δ-knowledge | 0.063 | (-0.306, 0.432) | 0.738 |  | 0.083 | (-0.285, 0.451) | 0.658 |  |
|  | Δ-self-care | 0.220 | (-0.311, 0.750) | 0.418 |  | 0.262 | (-0.264, 0.788) | 0.330 |  |
| **Δ-Cholesterol** | |  |  |  |  |  |  |  |  |
|  | Education level* | 10.288 | (-9.122, 29.698) | 0.300 |  | 9.370 | (-10.473, 29.213) | 0.355 |  |
|  | SES | 0.008 | (-0.102, 0.118) | 0.883 |  | 0.002 | (-0.108, 0.112) | 0.971 |  |
|  | Diabetes knowledge (baseline) | 1.323 | (-0.548, 3.195) | 0.167 |  | 1.395 | (-0.536, 3.325) | 0.158 |  |
|  | Diabetes knowledge (final) | 0.544 | (-1.801, 2.890) | 0.650 |  | 0.458 | (-1.997, 2.913) | 0.715 |  |
|  | Self-care (baseline) | 0.903 | (-2.015, 3.821) | 0.545 |  | 1.036 | (-1.919, 3.991) | 0.493 |  |
|  | Self-care (final) | 3.661 | (-0.480, 7.802) | 0.084 |  | 3.755 | (-0.443, 7.954) | 0.080 |  |
|  | Δ-knowledge | -0.901 | (-2.701, 0.900) | 0.328 |  | -0.986 | (-2.807, 0.835) | 0.289 |  |
|  | Δ-self-care | 0.705 | (-1.874, 3.285) | 0.592 |  | 0.625 | (-1.968, 3.217) | 0.637 |  |
| *Dichotomized (null vs the rest of categories). SES: Socioeconomic status. **Models adjusted for age (continuous), sex (categorical), and years of disease (continuous). | | | | | | | | | |
