# Supplementary material for: Role of social and other determinants of health in the effect of a multicomponent integrated care strategy on type 2 diabetes mellitus
Source: Int J Equity Health. 2020 May 24;19:75. doi: 10.1186/s12939-020-01188-2 (PMC7245830; doi:10.1186/s12939-020-01188-2)
Supplement: Supplementary file 2 — Additional file 2: Table S2. Association between social determinants of health, diabetes knowledge, and self-care in successfully reaching the triple target in DIABEMPIC participants (n = 498). [file 12939_2020_1188_MOESM2_ESM.docx]

| **Supplementary Table 2.** Association between social determinants of health, diabetes knowledge, and self-care in successfully reaching the triple target in DIABEMPIC participants (n=498). | | | | | | | | | | | |
| --- | --- | --- | --- | --- | --- | --- | --- | --- | --- | --- | --- |
|  |  | Univariable | | | |  | | Multivariable | | | |
|  |  | β | 95% CI | *p*-value |  | | β | | 95% CI | *p*-value |  |
| **Triple target (baseline)** | | |  |  |  | |  | |  |  |  |
|  | Education level* | 0.019 | (-0.035, 0.073) | 0.484 |  | | 0.014 | | (-0.041, 0.069) | 0.625 |  |
|  | SES | 0.000 | (-0.001, 0.001) | 0.454 |  | | 0.000 | | (-0.001, 0.001) | 0.456 |  |
|  | Diabetes knowledge (baseline) | 0.003 | (-0.002, 0.008) | 0.228 |  | | 0.003 | | (-0.002, 0.008) | 0.237 |  |
|  | Diabetes knowledge (final) | 0.001 | (-0.005, 0.008) | 0.654 |  | | 0.001 | | (-0.006, 0.007) | 0.852 |  |
|  | Self-care (baseline) | 0.007 | (-0.001, 0.014) | 0.084 |  | | 0.007 | | (-0.001, 0.14) | 0.088 |  |
|  | Self-care (final) | 0.005 | (-0.005, 0.016) | 0.326 |  | | 0.005 | | (-0.006, 0.016) | 0.362 |  |
|  | Δ-knowledge | -0.002 | (-0.007, 0.003) | 0.403 |  | | -0.002 | | (-0.007, 0.002) | 0.323 |  |
|  | Δ-self-care | -0.003 | (-0.010, 0.004) | 0.341 |  | | -0.003 | | (-0.010, 0.004) | 0.334 |  |
| **Triple target (final)** | |  |  |  |  | |  | |  |  |  |
|  | Education level* | -0.064 | (-0.240, 0.113) | 0.481 |  | | -0.087 | | (-0.266, 0.093) | 0.344 |  |
|  | SES | -0.001 | (-0.002, 0.001) | 0.218 |  | | -0.001 | | (-0.002, 0.001) | 0.151 |  |
|  | Diabetes knowledge (baseline) | 0.001 | (-0.015, 0.018) | 0.861 |  | | 0.002 | | (-0.015, 0.019) | 0.794 |  |
|  | Diabetes knowledge (final) | 0.018 | (-0.003, 0.039) | 0.102 |  | | 0.017 | | (-0.004, 0.039) | 0.117 |  |
|  | Self-care (baseline) | -0.016 | (-0.041, 0.009) | 0.204 |  | | -0.015 | | (-0.040, 0.010) | 0.235 |  |
|  | Self-care (final) | 0.009 | (-0.027, 0.046) | 0.608 |  | | 0.010 | | (-0.026, 0.046) | 0.587 |  |
|  | Δ-knowledge | 0.009 | (-0.007, 0.024) | 0.289 |  | | 0.007 | | (-0.009, 0.023) | 0.366 |  |
|  | Δ-self-care | 0.017 | (-0.006, 0.040) | 0.139 |  | | 0.016 | | (-0.006, 0.039) | 0.156 |  |
| *Dichotomized (null vs the rest of categories). SES: Socioeconomic status. **Models adjusted by age (continuous), sex (categorical) and years of disease (continuous). | | | | | | | | | | | |
